# Cytidine deaminase enzymatic activity is a prognostic biomarker in gemcitabine/platinum-treated advanced non-small-cell lung cancer: a prospective validation study

**DOI:** 10.1038/s41416-018-0307-3

**Published:** 2018-11-08

**Authors:** Carmelo Tibaldi, Andrea Camerini, Marcello Tiseo, Francesca Mazzoni, Fausto Barbieri, Isabella Vittimberga, Matteo Brighenti, Luca Boni, Editta Baldini, Annalisa Gilli, Richard Honeywell, Myriam Chartoire, Godefridus J. Peters, Elisa Giovannetti, on behalf of The Italian Oncological Group of Clinical Research (GOIRC)

**Affiliations:** 1 Department of Oncology, S. Luca Hospital, Lucca, Italy; 2 Department of Oncology, Versilia Hospital, Lido di Camaiore, Camaiore, Italy; 3 Department of Oncology, Azienda Ospedaliero-Universitaria, Parma, Italy; 4 Department of Oncology, Azienda Ospedaliero-Universitaria, Firenze, Italy; 5 Department of Oncology, Azienda Ospedaliero-Universitaria, Policlinico of Modena, Modena, Italy; 6 Department of Oncology, Lecco, Italy; 7 Department of Oncology, ASST of Cremona, Cremona, Italy; 8 Clinical Trials Coordinating Center, Istituto Toscano Tumori, Azienda Ospedaliero-Universitaria, Firenze, Italy; 9 Department of Medical Oncology, Amsterdam University Medical Center, VU University, Amsterdam, The Netherlands

**Keywords:** Medical research, Non-small-cell lung cancer, Predictive markers

## Abstract

**Background:**

Cytidine deaminase (CDA) plays a crucial role in the degradation of gemcitabine. In our previous retrospective study, CDA enzymatic activity was the strongest prognostic biomarker of the activity and efficacy of platinum/gemcitabine combinations. The aim of this prospective study was to validate the prognostic role of CDA activity in the first-line treatment of advanced non-small-cell lung cancer.

**Methods:**

A total of 124 untreated patients received standard doses of platinum/gemcitabine. CDA activity was baseline measured in plasma samples by spectrophotometric assay.

**Results:**

Using the median CDA level as cut-off, in the patients with high versus low CDA activity the response rate was 25.0% (95% CI, 14.7–37.8) and 54.1% (95% CI, 40.8–66.9), *P* = 0.0013; the 6-month progression rate was 34.5% (95% CI, 22.6–46.6) and 54.1% (95% CI, 40.9–65.6), HR = 2.01 (95% CI, 1.32–3.06), *P* < 0.001; the 1-year survival rate was 23.3% (95% CI, 13.6–34.6) and 57.3% (95% CI, 43.9–68.6), HR = 2.20 (95% CI, 1.46–3.34), *P* = 0.0002, respectively. CDA activity resulted to be an independent prognostic factor for progression and survival at multivariate analysis.

**Conclusions:**

This study validated prospectively the prognostic role of the CDA activity and should prompt larger and adequately designed randomised prospective studies to establish the predictive impact of this test in improving the outcome of selected patients.

## Introduction

Non-small-cell lung cancer (NSCLC) is the leading cause of cancer death, with only 1% 5-year survival rate for stage IV disease.^1^ The current standard of care in first-line advanced NSCLC, which does not express targetable oncogene driver alterations, or has a PD-L1 expression level< 50%, is a platinum compound combined with a last-generation therapeutic agent, most commonly taxanes combined or not with bevacizumab, gemcitabine, vinorelbine or pemetrexed.^2^ However, therapy with platinum-based doublets has reached a therapeutic plateau.^3^

Platinum/gemcitabine combination is one of the most commonly used regimens in clinical practice,^2^ but inter-individual variability in clinical response and efficacy has been observed. In this context, the discovery of biomarkers with predictive power should be warranted to assess inter-patient differences in clinical outcome.

The enzyme cytidine deaminase (CDA) plays a key role in the metabolism of gemcitabine (2′,2′-difluorodeoxycytidine) to its inactive metabolite, 2′,2′-difluorodeoxyuridine (dFdU).

In our previous multicenter retrospective clinical studies, we analysed CDA polymorphisms^4,5^ and CDA enzymatic activity^5^ in 126 advanced NSCLC patients treated with platinum/gemcitabine. We observed that CDA enzymatic activity, measured in blood samples by high-performance liquid chromatography (HPLC), was the strongest prognostic biomarker of activity and efficacy of this combination.^5^ By using the median distribution level of CDA activity as cut-off, patients with low CDA activity had higher response rate (37.7% versus 13.8%; *P* = 0.006), longer time to progression (8.0 versus 3.0 months; *P* < 0.001) and longer overall survival (OS, 19.0 versus 6.0 months; *P* < 0.001) than patients with high CDA activity.^5^

Additionally, a retrospective pivotal study suggested that CDA functional testing identified CDA deficient patients likely to experience severe toxicities with gemcitabine.^6^

However, the determination of CDA activity by the HPLC method is a time-consuming and relatively expensive process.

Apart from HPLC, another method to measure CDA enzymatic activity is a spectrophotometric assay.^7^

An EORTC-PAMM collaborative initiative compared these two techniques and demonstrated that the determination of the CDA enzymatic activity via a spectrophotometric method was the most simple and cost-effective validated test for therapeutic monitoring purposes.^8^ The aim of this prospective multicenter study was to validate the prognostic role of CDA enzymatic activity determined by spectrophotometric assay in terms of activity and efficacy in advanced NSCLC patients treated with a platinum/gemcitabine combination as first-line treatment. Conversely, since CDA enzymatic activity emerged as the strongest prognostic biomarker, we performed no further genotype-to-phenotype studies.

## Patients and methods

### Patients

Our study involved eight Italian medical Oncology Units. Chemotherapy-naive patients with histologically or cytologically proven NSCLC without activating EGFR mutations were enrolled in the study. Study entry was limited to the patients aged ≥ 18 years of age, with Eastern Cooperative Oncology Group (ECOG) performance status (PS) 0–1 and life expectancy >12 weeks and with measurable clinical stage IIIB or IV disease. Adequate bone marrow, as well as renal and liver function were required. Patients with brain metastases were eligible for trial participation if they were adequately treated and neurologic findings had returned to baseline. Exclusion criteria included other (previous or current) active malignancies, active infections, recent myocardial infarction, unstable angina.

The study was approved by the local Hospital Ethics Committees and conducted according to the Good Clinical Practice Guidelines and to the *World Medical Association* Helsinki declaration.

Informed consent for this study and related blood samples were obtained before chemotherapy treatment.

### Evaluation criteria

Pretreatment evaluation included medical history, physical examination, complete blood cell count with routine chemistry and computed-tomography (CT) scan of chest and abdomen.

Tumour response was evaluated by CT scan every three cycles. Responses were assessed using RECIST criteria version 1.1, and the best overall response was reported for each patient. After the end of treatment, tumour radiological evaluation according to RECIST criteria was carried out every 2 or 3 months until evidence of progressive disease.

Haematological and non-haematological toxicities were recorded at days 1 and 8 of every treatment course. The worst toxicity grade was reported for all chemotherapy cycles. Toxicities were assessed by using the National Cancer Institute common terminology criteria (*NCI-CTC* 3.0 version).

### Treatment

Chemotherapy consisted in cisplatin 80 mg/m^2^ infused over 60 min on day 1 and gemcitabine 1200 mg/m^2^ was administered intravenously over 30 min on days 1 and 8, or carboplatin AUC-5 infused over 60 min on day 1 and gemcitabine 1000 mg/m^2^ administered intravenously over 30 min on days 1 and 8; both regimens for a maximum of 6 courses every 3 weeks. Treatment was discontinued in the case of progression of disease, major toxicities or according to the patient’s or physician’s decision.

The criteria for dose reductions and treatment delay are reported in the Supplementary data.

### Samples

Blood samples were obtained from each patient at baseline and stored at −80 °C. In nine cases, we collected the plasma both at baseline and at the beginning of the second course. Additional studies on cancer cell lines, xenograft and tumour tissues are reported in the Supplementary data.

### Analysis of CDA enzymatic activity

CDA activity was measured in plasma samples by a validated spectrophotometric assay, by using an absorbance plate reader operated with Gen 5v.2 software, while protein concentration was evaluated with the bicinchoninic colorimetric assay, as described previously.^6,7^ The general principle of this assay is to evaluate a CDA-catalysed deamination reaction, which converts cytidine to uridine with a stoichiometric release of an ammonium ion. The ammonium ion can be coupled in a second step with phenol. The absorbance is then determined by using visible spectrophotometry at a wavelength of 625 nm. We believe that this spectrophotometric assay to measure CDA activity is a simple, cost efficient, and reproducible test that could be performed in community hospitals and clinics following the protocol detailed in the Supplemental Methods.

The patients were grouped according both to the median distribution level of CDA enzymatic activity, as established in our previous study,^5^ and as confirmatory analysis of sensibility to cut-off for the optimal distribution level of CDA enzymatic activity, calculated by the Contal & O’Quigley test.^8^ Analysis of all the samples was performed in blinded fashion and related to clinical outcome.

### Statistical analysis

By assuming a response rate equal to 14% in the group of patients with high CDA enzymatic activity and balanced distribution of the enzymatic status, an overall sample size of 104 patients guaranteed to the study a power of 80%, for a two-sided chi-square test for heterogeneity and an alpha-error equal to 5%, in favour of the hypothesis of a response rate equal to or greater than 40% in the group of patients with low CDA enzymatic activity. The sample size was increased to 124 patients, to avoid the reduction power due to any unbalance in the distribution of the enzymatic status. These allowed to observe a number of events (71) that showed, with a power equal to 80%, a relative reduction in the risk of disease progression or death of at least 50% (HR = 0.50) in patients with low CDA enzymatic activity versus patients with high CDA enzymatic activity (two-sided log-rank test with an alpha error equal to 5%). It was assumed that this goal could be achieved 6–8 months after enrolment of the last study patient.

Logistic regression was conducted to determine the association between CDA activity and therapy responsiveness, by calculating the odds ratio (OR) with 95% confidence intervals (CI).

The life table method was used to plot progression-free survival (PFS) and OS, and the log-rank test was employed to compare curves in univariate analysis. The treatment hazard ratio (HR) for progression or death, and its 95% CI, was estimated with using the likelihood ratio test of the model.

The prognostic variables of PFS and OS in univariate analysis were included in the multivariate analysis by using Cox’s proportional hazard model.

## Results

### Patients’ characteristics

From March 2013 to March 2016 a total of 124 consecutive Caucasian patients, affected by advanced NSCLC, were enrolled in the study and 121 patients were analysed for CDA activity and were evaluable for final analysis. The majority of patients had stage IV (78.5%) disease, while 26 (21.5%) had stage IIIB disease (clinical characteristics are reported in Table 1). Median follow-up for living patients was 36 months (range, 22–38 months). The overall response rate was 39.6% (95% CI, 30.8–48.9). According to the life table method, the progression rate at 6 months was 44.5% (95% CI, 35.5–53.2) and the survival rate at 1-year was 40.4% (95% CI, 31.6–49.0). Median PFS and OS estimated by the Kaplan–Meier method were 5.0 (95% CI, 4.4–5.6) and 10.0 (95% CI, 8.4–11.6) months, respectively.

**Table 1:** Clinical characteristics

Characteristics	Patients, *n* (%)
No. patients	121
*Age, median years*	70
Range	49–87
*Sex*	
Male	94 (77.6)
Female	27 (22.4)
*Clinical stage*	
IIIB	26 (21.5)
IV	95 (78.5)
*ECOG PS*	
0	48 (39.7)
1	64 (52.9)
2	9 (7.4)
*Histology*	
Adenocarcinoma	28 (23.1)
Epidermoid	75 (62.0)
Large cells	18 (14.9)
*Therapy*	
CDDP-Gem	48 (39.7)
CBDCA-Gem	73 (60.3)

*CDDP* cisplatin, *CBDCA* carboplatin, *GEM* gemcitabine, *ECOG* Eastern Cooperative Oncology Group, *PS* performance status

**Fig. 1:**
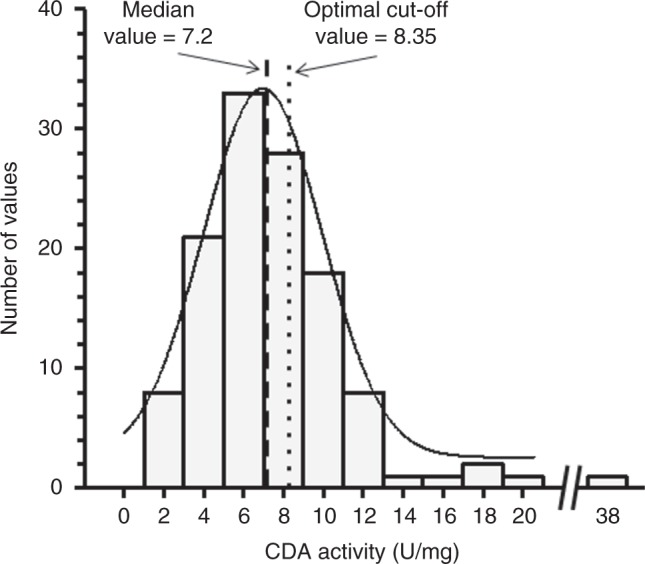
Distribution of the CDA activity values. The CDA activity values showed a Gaussian/normal distribution among the NSCLC patients enrolled in the present study. Statistical analysis was performed with the one sample Kolmogorov–Smirnov test, also called the Kolmogorov–Smirnov goodness-of-fit test, showing that this distribution passed the normality test (alpha = 0.05) with a *P* = 0.20, while skewness and kurtosis were 1.124 and 2.237, respectively. The dashed and pointed lines indicate the two cut-offs (CDA median value and optimal cut-off, respectively)

The median distribution and the optimal distribution of cut-off levels of CDA enzymatic activity in all 121 patients was 7.2 U/mg (range, 1.73–37.5) and 8.35 U/mg (range, 1.73–37.5), respectively (Fig. 1). Patients’ characteristics according to median CDA value and optimal CDA value are reported in the supplementary Tables S1 and S2, respectively.

### Correlation between enzymatic activity and overall response rate

By using the median CDA distribution level as cut-off, patients with low CDA activity had an overall response rate of 54.1% (95% CI, 40.8–66.9), whereas patients with high CDA activity reported an overall response rate of 25.0% (95% CI, 14.7–37.8); OR = 0.28 (95% CI, 0.13–0.61); *P* = 0.0013.

Univariate analysis of the overall response rate demonstrated a correlation with performance status (*P* = 0.0095), type of platinum (*P* = 0.0095), CDA activity (*P* = 0.0015); and a trend towards a significant association was observed for the histotype (*P* = 0.059). Multivariate analysis did not confirm the prognostic significance of CDA activity (see Supplementary Table S3). However, this was the result of an abnormal distribution of favourable prognostic factors in the low CDA activity group of patients due to chance (see Supplementary Table S1). Importantly, if the optimal distribution level of CDA activity was used for the cut-off point, multivariate analysis then showed a significant independent prognostic association between CDA assay and treatment activity (see Supplementary Results and Table S4).

### Correlation between enzymatic activity and clinical outcome

**Fig. 2:**
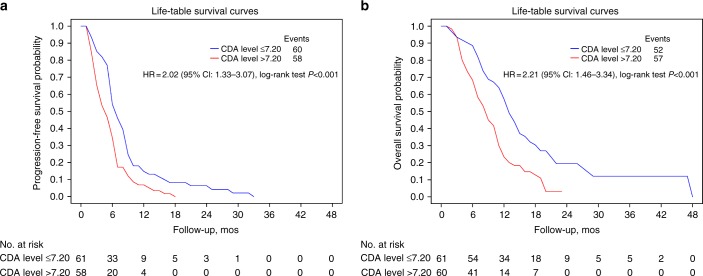
Correlation of CDA activity with outcome. Curves for progression-free survival (PFS, panel **a**) and overall survival (OS, panel **b**) according to CDA activity, using as cut-off the CDA median value. The life table method was used to plot PFS and OS, as explained in the Methods

Considering the median distribution level of CDA activity as the cut-off point, we observed a progression rate at 6 months of 34.5% (95% CI 22.6–46.6) in the group of patients with high CDA activity compared to 54.1% (95% CI 40.9–65.6) in the group of patients with low CDA activity, HR = 2.01 (95% CI 1.32–3.06); *P* < 0.001 (Fig. 2, panel a).

The 1-year survival rate was 23.3% (95% CI 13.6–34.6) in the group of patients with high CDA activity and 57.3% (95% CI 43.9–68.6) in the group of patients with low CDA activity, HR = 2.20 (95% CI 1.46–3.34); *P* = 0.0002 (Fig. 2, panel b).

The Cox proportional hazards regression model used for multivariate analysis confirmed CDA enzymatic activity as independent prognostic factor for progression and survival (Table 2, panel A, B). Additionally, we obtained similar but more pronounced differences by using the optimal distribution level of CDA activity as cut-off (see Supplementary data, Table S5, Figure S1).

**Table 2:** Multivariate analysis of CDA on progression-free survival (panel A) and overall survival (panel B) (cut-off 7.2 U/mg)

Parameter	DF	Parameter estimate	Standard error	Chi-square	Pr > Chisq	Hazard ratio	95% Confidence limits
Panel A							
Age	1	−0.01623	0.01491	1.1847	0.2764	0.984	0.956–1.013
Sex: female	1	0.55047	0.28231	3.8020	0.0512	1.734	0.997–3.016
ECOG PS 1	1	0.50064	0.23169	4.6691	0.0307	1.650	1.048–2.598
ECOG PS 2	1	1.20025	0.46382	6.6964	0.0097	3.321	1.338–8.243
Stage IIIB	1	−0.49090	0.27448	3.1987	0.0737	0.612	0.357–1.048
CDA high > 7.2	1	0.47659	0.22985	4.2993	0.0381	1.611	1.026–2.527
Panel B							
Age	1	−0.01091	0.01466	0.5536	0.4569	0.989	0.961–1.018
Sex: female	1	0.18483	0.25073	0.5434	0.4610	1.203	0.736–1.967
ECOG PS 1	1	0.64678	0.22697	8.1206	0.0044	1.909	1.224–2.979
ECOG PS 2	1	0.98242	0.41952	5.4839	0.0192	2.671	1.174–6.078
Stage IIIB	1	−0.81212	0.28671	8.0236	0.0046	0.444	0.253–0.779
CDA high > 7.2	1	0.58208	0.22099	6.9376	0.0084	1.790	1.161–2.760

*ECOG* Eastern Cooperative Oncology Group, *PS* performance status, *CDA* cytidine deaminase

### Comparison between enzymatic activity analysis performed by HPLC and spectrophotometric assay

**Fig. 3:**
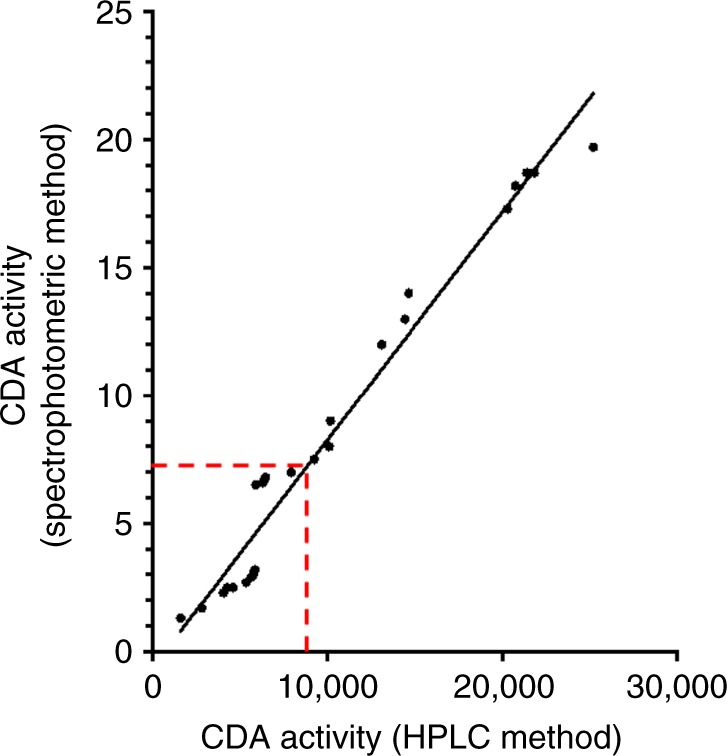
Comparison between CDA activity analysis methodologies. Values of CDA activity analysis obtained by HPLC and spectrophotometric assays in 30 randomly selected samples. Linear regression was calculated by Graph Pad Prism (version 7)

In order to compare the HPLC assay used in our previous clinical study (5) and the spectrophotometric assay used in this study, we performed parallel analyses in 30 plasma samples by using both the HPLC and the spectrophotometric assays, as described previously (7). The CDA activity measured with both assays was linear in time and with the amount of protein added. The correlation between the results obtained using these methodologies was excellent (linear regression: *r*^2^ = 0.923, *P* < 0.00001), with 100% of samples identified as low or high by both tests (see Fig. 3). Similar results were observed in cancer cell lines and the median CDA plasma activity was consistent with previously published results, as reported in the Supplementary data.

### Evaluation of intra-individual longitudinal variability of CDA activity

In eight patients we performed an exploratory analysis of intra-individual longitudinal variability of CDA activity by means of plasma samples obtained at baseline and at the beginning of the second cycle. In all these cases we observed similar or reduced values of CDA activity. However, as shown in Figure S2, in most cases the reduction was minimal (on average 10%) and did not change the subgrouping of the patients (four patients were still in the “low CDA activity group” and three patients remained in the “high CDA activity group”). In contrast, in one patient we observed an almost threefold reduction of CDA activity levels; while at baseline this patient was categorised in the “high CDA activity” group, after treatment the same patient would be included among the patients with “low CDA activity”. This patient had a partial response, and we could speculate that the sample obtained after treatment could better predict his clinical outcome. However, further studies are needed to understand whether samples taken at different time-points might improve the prognostic/predictive value of baseline CDA activity; and whether different factors such as changes in the chemotherapy regimen and/or clinicopathological features might be monitored together with CDA activity.

### Toxicities

All 121 patients were evaluable for toxicity and received a total of 490 cycles.

Patients with low CDA activity received 265 cycles, while those with high CDA activity received 225 cycles; the median number of cycles was 4 (range, 1–6) in both groups.

Fifty-four (20.3%) out of 265 courses in the group of patients with low CDA activity and 43 (19.1%) out of 225 courses in the group of patients with high CDA activity were delayed because of toxicity (*P* = 0.72).

The frequency of dose reduction was 26.8% (71/265) of courses in the group of patients with low CDA activity and 28.4% (64/225) of courses in the group with high CDA activity (*P* = 0.68).

The toxicities reported in the subgroups of patients with differential activity are listed in Table 3. No significant association was observed between level of CDA activity and severe toxicities. No treatment-related deaths occurred.

**Table 3:** Toxicity according to CDA activity (number of patients)

	Low activity ≤7.2 U/mg (61 pts)	High activity > 7.2 U/mg (60 pts)
	Grade 3–4 (%)	Grade 3–4 (%)
Haematological (all events)	18 (29.5)	13 (22)
Anaemia	6 (9.8)	5 (8.3)
Neutropenia	20 (32)	16 (26.6)
Thrombocytopenia	9 (14.7)	7 (11.6)
Non-haematological (all events)	10 (16)	10 (17)
Vomiting	4 (6.5)	3 (5)
Nausea	3 (4.9)	4 (6.6)
Weakness	3 (4.9)	3 (5)
Paraesthesia	0	0
Tinnitus	0	0
Hepatic toxicity	0	0
Renal toxicity	0	0

Most patients had several haematological toxicities and only the worst toxicity grade among all the toxicities for each patient was reported

NOTE. Data on haematological and non-haematological toxicity were available from 121 patients. Considering the total number of toxicities in each CDA subgroup, no significant association was observed with the Pearson-χ^2^ two-sided test with continuity correction.

## Discussion

This multicenter study evaluated the impact of CDA enzymatic activity on outcomes of advanced NSCLC patients treated with first-line platinum–gemcitabine regimens and prospectively validated the prognostic role of CDA activity as independent prognostic biomarker of activity and efficacy in these patients. We therefore confirmed the hypothesis deriving from our previous multicenter retrospective study^5^ according to which patients with low baseline CDA enzymatic activity could double the response rate and a relative reduction in the risk of disease progression or death of at least 50% compared to patients with high CDA enzymatic activity. As established in our previous study,^5^ the patients were grouped according to the median distribution level of CDA enzymatic activity. In addition, as confirmatory analysis of sensibility, we also used the cut-off for the optimal distribution level of CDA enzymatic activity, calculated by the Contal & O’Quigley test,^8^ which gave similar results.

Although most advanced NSCLC patients currently receive chemotherapy, there is no validated biomarker routinely used in clinic practice to customise chemotherapy treatment.^9^

The recently published ERCC1-trial (ET) was the largest prospective randomised phase-III study in advanced NSCLC specifically designed to evaluate prospective testing of ERCC1 in the tumour tissue with immunohistochemistry assay, as predictive biomarker of platinum-based chemotherapy.^10^ ET failed to demonstrate any predictive role of the ERCC1 test evaluated either alone or in combination with the XPF assay.

In line with these results was a previous phase-III trial that failed to demonstrate any differences in PFS between carboplatin/gemcitabine and a customised chemotherapy consisting in carboplatin/gemcitabine, carboplatin/docetaxel, gemcitabine/docetaxel or docetaxel/vinorelbine according to the ERCC1 and RRM1 tumour protein levels.^11^ Several elements may have contributed to the negative results generated by these studies,^12^ such as the power of biomarkers investigated that was insufficient to detect clinically significant differences; the absence of reproducible and analytically validated assays and the heterogeneity of tumour tissue and/or the cellular context that may have negatively impacted the results.

We hypothesise that the correlation of CDA activity with efficacy outcome (OS and PFS) might be the result of differential detoxification of gemcitabine by both the liver and the tumour cells. The advantage of CDA enzymatic assay lies in the fact that it is not affected by limited material availability, nor by intra- and inter-patient heterogeneity of cancer disease, thus reducing the variability of test results. The spectrophotometric assay was validated within an EORTC-PAMM collaborative initiative as a very simple and cost-effective test that only required basic bench apparatus and standard reagents, making it easily transportable to any laboratory.^7^ All these characteristics are important for the clinical feasibility of a potential biomarker.

In the pivotal study by Ciccolini and collaborators, although without cases of toxic deaths or hospital admission for toxicity, patients with low CDA activity experienced an early severe haematological toxicity.^6,13^ Despite the wide variability of CDA activity between 1.73 and 37.5 U/mg, we observed no significant differences in terms of severe toxicity between the group of patients with low CDA activity and the group of patients with high CDA activity. However, in the study performed by Ciccolini and collaborators,^6^ the CDA activity cut-off statistically associated with the event of early severe toxicity was 1.3 U/mg. In our study, only one patient had a baseline CDA enzymatic activity < 2 U/mg (1.73 U/mg). This patient did not experience any early severe toxicity and received a total of 6 courses of chemotherapy.

Another likely explanation for this observation could be that in this study the haematological and non-haematological toxicity was recorded only on days 1 and 8 of every course of chemotherapy. Severe haematological toxicity, which eventually occurred on day −15, was not recorded among the clinical data collected and therefore we could not correlate these data with CDA activity. All our patients uniformly received platinum/gemcitabine as first-line treatment and for this reason were at lower risk of toxicity compared to heavily pretreated patients enrolled in the study performed by Ciccolini and collaborators. Moreover, in this last study patients received different types of gemcitabine-based chemotherapy regimens, so that the variability of results increased.

Serum CDA activity has been evaluated as a biomarker of inflammatory activity in different inflammatory diseases, including rheumatoid arthritis and gout.^14,15^ These findings support further studies performing parallel measurements of biomarkers of inflammation, such as C-reactive protein (CRP). However, elevated levels of CRP have been correlated with tumour size and staging of NSCLC, and a meta-analysis demonstrated that an elevated CRP level is associated with poorer survival of NSCLC patients and might be used as a prognostic biomarker.^16^ In the present study, we did not measure CRP in the same samples that were withdrawn to evaluate CDA status and we could not evaluate the potential correlation between these biomarkers. Future studies should investigate the role of inflammation in the modulation of CDA activity, as well as intra-individual longitudinal variability. In the present study, we were able to report only a minimal variation of CDA activity in plasma samples collected at baseline and at the beginning of the second cycle. However, in one patient, we observed a significant reduction of CDA activity, which could have a clinical impact, and should be further investigated.

Our analysis is limited by the small sample size of our population. Large-scale validation, including a matched cohort of patients treated with another regimen, is needed to further verify the predictive potential of CDA. Secondly, the selected cut-off of CDA activity was deliberately based on the median value, as reported in our previous study. This focused approach, together with the established protocol ensured accurate statistics, but further optimisation, as performed by the Contal & O’Quigley test, might be critical for translation to the clinical setting. Finally, future studies should evaluate the modulation of CDA activity in inflammatory conditions as well as intra-individual variability of CDA activity over time.

Although the cisplatin–pemetrexed combination regimen has recently been preferred for the subgroup of NSCLC patients with no-squamous histology,^17^ the gemcitabine–platinum regimens still play a key role in chemotherapy for all patients with advanced NSCLC. Moreover, platinum/gemcitabine combinations are commonly used for the daily treatment of several other types of tumours other than NSCLC including nasopharynx cancer, ovarian carcinoma, triple negative breast cancer, bladder carcinoma, bile duct cancer, whereas gemcitabine combinations are commonly used in pancreatic carcinoma and in soft tissue sarcomas.^18^ Therefore, we can hypothesise other possible future clinical applications of the present test, in case this biomarker is validated as predictive biomarker, guiding the choice of an alternative chemotherapeutic regimen and/or the modulation of the dose of gemcitabine according to CDA activity in NSCLC and in other tumours.

For these reasons, after the present prospective study, we are currently working with the PAMM-EORTC group, the European Medicines Agency and national/regional Health Authorities on defining the best strategy to be undertaken at bedside to implement the use of the CDA activity test in clinical practice. In particular, the planning of a randomised prospective phase-III trial with a control arm of patients treated with another regimen and the comparison of the clinical outcome stratified by CDA activity levels would be critical to definitely establish the predictive role of CDA activity for patients treated with gemcitabine-based regimens. This future trial should follow a standardised study design and include appropriate power calculations of the sample size for the specific predictive factor, in order to have adequate statistical power.

## Electronic supplementary material

Supplementary data

Supplementary Figure 1

Supplementary Figure 2

Supplementary Figure 3

Supplementary Table 1

Supplementary Table 2

Supplementary Table 3

Supplementary Table 4

Supplementary Table 5A-B

## Availability of data and material

Data and material are available upon request by Department of Oncology, Lucca, Italy.
