# Supplementary material for: Cytidine deaminase enzymatic activity is a prognostic biomarker in gemcitabine/platinum-treated advanced non-small-cell lung cancer: a prospective validation study
Source: Br J Cancer. 2018 Nov 8;119(11):1326–31. doi: 10.1038/s41416-018-0307-3 (PMC6265283; doi:10.1038/s41416-018-0307-3)
Supplement: Supplementary file 1 — Supplementary data [file 41416_2018_307_MOESM1_ESM.doc]

**Supplementary data**

**Methods**

**Treatment.**

Patients whose white blood cell count, neutrophil count and platelet count were greater than 3.5 x10^9^/L, 1.5x10^9^/L and 100x10^9^/L, respectively, received chemotherapy on day 1. If these conditions were not met, administration of chemotherapy was delayed for one week or until recovery. A dose delay for more than 3 weeks resulted in withdrawal from the study. The dose of gemcitabine was modified on day-8 according to haematological and non-haematological toxicities as follows: if neutrophil count was >1.5x10^9^/L and platelet count was >100x10^9^/L, chemotherapy was administered at full dose; for neutrophil count 1.0-1.49x10^9^/L or platelet count 75-99x10^9^/L, the dose was reduced to 75%; for neutrophil count 0.5-0.9x10^9^/L or platelet count 50-74.9x10^9^/L the dose was reduced to 50%; for neutrophil count <0.5x10^9^/L or platelet count <50x10^9^/L chemotherapy was omitted. In case of grade-2 non-haematological toxicity (except for alopecia) chemotherapy was omitted until resolution, and then administered in the next cycle at doses reduced by 25%. In case of grade-2 neurological toxicity or grade-3-4 non-haematological toxicity the patient was withdrawn from the study.

**Analysis of CDA enzymatic activity.** The CDA spectrophotometric assay was performed using the following detailed chemicals and protocol:

**Chemicals.** The working buffer solution (pH=7) was composed of potassium dihydrogen phosphate (Merck Laboratory, batch: A850773) and Di-sodium hydrogen phosphate (Merck laboratory, 247A713186) with a minimum purity of 99%. The substrate solution was cytidine (C9505), purchased from Sigma-Aldrich laboratory (B.V, the Netherlands) with a purity of 99 %. Phenol (CAS number: 108-95-2) was purchased from AMRESCO laboratory (Ohio, USA) with purity upper than 99%. Sodium tungstate (SZB81370) was purchased from Sigma-Aldrich laboratory (B.V, the Netherlands) with a minimum purity of 99%. Sodium hypochlorite (Product number: 425044) was purchased from Sigma-Aldrich laboratory (B.V, the Netherlands) with purity between 10 and 15%. Sodium nitroprusside dihydrate (product number: 50501) was purchased from Sigma-Aldrich laboratory (B.V, the Netherlands) with purity between 99 and 102%. Sulfuric acid (012k13881131) was purchased from Merck laboratory (B.V, the Netherlands) with purity between 95 and 97%. Ammonium chloride (H4696) was purchased from J.T. Baker laboratory (B.V, the Netherlands) with a purity of 99.8%. Protein analysis was performed using the bicinchoninic colorimetric assay supplied by Thermofisher scientific B.V, Breda, the Netherlands (catalogue number: 23225).

**Protocol.** Briefly, in a 96-wells plate a 7-point calibration line (40, 20, 10, 5, 2.5, 1.25, and 0) for ammonium chloride in 20 μl of buffer and 20 μl of plasma was combined with 80 μl 2 mM cytidine (final 1.6 mM), and incubated for 16 hours incubation at 37◦C. Each sample was evaluated at least in duplicate. The plate configuration included three different dilutions for each sample. After ultrasonic bath and centrifugation of samples, dilution 1:10 (green wells), 1:30 (orange wells), 1:50 (yellow wells) were added in the plate. The border wells stayed empty, and parafilm was put on the plate before the overnight of incubation to prevent evaporation of the samples closest to the plate’s edges.

Ammonia was measured spectroscopically after the addition of 40 μl sodium tungstate and 40 μl 1N sulphuric acid. The plates were centrifuged and 25 μl was transferred to another plate where 75 μl phenol solution and 100 μl hypochlorite solution were added. The absorbance was measured at 625 nm, as reported previously (Peters et al., 2014). Activity was expressed as arbitrary unit (AU)/mg protein, which represents pmol ammonia/hr/mg protein. All data points not conforming to established specifications (<blank, >top calibration and duplicates ±15%) were discarded. For each plate, one calibration curve and one table were obtained with the concentration and the absorbance of the different samples. To validate the standard curve, the linear regression coefficient (R2) was never lower than 0.98.

**Analysis of CDA enzymatic activity in cancer cells, xenografts and fresh tumour tissues.** All the patients enrolled in the present study were affected by advanced NSCLC and we did not have specimens that could be used to evaluate CDA activity in the tumour. However, in order to compare CDA enzymatic activity in plasma with other specimens, we performed additional analyses in NSCLC cancer cells and xenografts (H460, Calu-6, Calu-1 and A549) as well as in a group of 12 fresh esophageal tumours (obtained by resected patients through a protocol approved by the appropriate Ethical Committee at VUmc, Amsterdam, The Netherlands) using spectrophotometric or HPLC analysis.

**Statistical Analysis.** Progression-free survival (PFS) was defined as the time interval between the date of enrolment in the study and the date of progression/death or the last-known progression-free date (censored). Overall survival (OS) was defined as the time interval between the date of enrolment in the study and the date of death due to any cause or last follow-up (censored).

**Results**

**Correlation between enzymatic activity and overall response rate.**  Using as cut-off the optimal CDA distribution level, patients with low CDA activity had an overall response rate of 50.0% (95% CI, 38.1-61.8) whereas patients with high CDA activity reported an overall response rate of 23.4% (95% CI 12.3%-38.0), OR= 0.306, (95% CI, 0.13-0.69); P=0.0043.

At the univariate analysis, Performance Status (P= 0.0095), histotype (P= 0.059), type of platinum (P= 0.0095) and CDA activity (P= 0.0042) resulted correlated with the overall response rate. The multivariate analysis confirmed the prognostic significance of CDA activity (P= 0.04) (see supplementary Table S4).

**Correlation between enzymatic activity and clinical outcome.** Applying as cut-off the optimal CDA distribution level, we observed a PFS at 6 months of 30.0% (95% CI 18.0-44.0) in the group of patients with high CDA activity compared to 53.1% (95% CI 41.0-64.0) in the group of patients with low CDA activity, HR= 2.29 (95% CI 1.48-3.55); P=0.0002.

The 1-year OS was 19.1% (95% CI 9.4-31.3) in the group of patients with high CDA activity and 53.9% (95% CI 41.9-64.5) in the group of patients with low CDA activity, HR= 2.4 (95% CI 1.58-3.68); P< 0.001 (Figure S1).
The Cox proportional hazards regression model used for the multivariate analysis confirmed CDA enzymatic activity being and independent prognostic factor for PFS and OS (Table S5A-B).

**Analysis of CDA activity in cancer cells, xenografts and tumour tissues.** These analyses showed a range of CDA values which was wider compared to the range observed in the plasma of the NSCLC patients (Figure S3). However, considering the different nature and low number of the tumour samples, we could not draw conclusions on the comparability of plasma and tumour values, and we conclude that further studies are needed to obtain deeper insights on the levels of CDA activity in different tissues.

**References** (in alphabetic order)

Peters GJ, Honeywell RJ, Maulandi M, Giovannetti E, Losekoot N, Etienne-Grimaldi MC, Milano G, Serdjebi C, Ciccolini J; EORTC-Pharmacology and Molecular Mechanism Group. Selection of the best blood compartment to measure cytidine deaminase activity to stratify for optimal gemcitabine or cytarabine treatment. Nucleosides Nucleotides Nucleic Acids. 2014;33(4-6):403-12.

Tibaldi C, Giovannetti E, Tiseo M, Leon LG, D'Incecco A, Loosekoot N, Bartolotti M, Honeywell R, Cappuzzo F, Ardizzoni A, Peters GJ. Correlation of cytidine deaminase polymorphisms and activity with clinical outcome in gemcitabine-/platinum-treated advanced non-small-cell lung cancer patients. Ann Oncol. 2012;23(3):670-7.

**Figure Legends.**

**Figure S1.** Life-table curves for progression-free Survival (Panel A) and overall Survival (Panel B), using as cut-off the optimal CDA distribution value.

**Figure S2.** Exploratory analysis of intra-individual longitudinal variability of CDA activity, considering samples obtained at baseline and at the beginning of the second cycle (after treatment) in eight randomly selected patients. The black lines connect the two samples of each patient, the dashed and pointed horizontal lines indicate the 2 cut-offs (CDA median value and optimal cut-off, respectively), while the red and blue dots identify the “high CDA” and “low CDA” activity groups, respectively.

**Figure S3.** CDA enzyme activity levels in cancer cells (black dots), xenografts (green dots) and tumour specimens (purple dots). The continuous horizontal lines indicate the CDA median value in these three different subgroups, while the dashed line indicate the CDA median value cut-off in the plasma samples (i.e., 7.2 U/mg protein).
