# Supplementary material for: Cytidine deaminase enzymatic activity is a prognostic biomarker in gemcitabine/platinum-treated advanced non-small-cell lung cancer: a prospective validation study
Source: Br J Cancer. 2018 Nov 8;119(11):1326–31. doi: 10.1038/s41416-018-0307-3 (PMC6265283; doi:10.1038/s41416-018-0307-3)
Supplement: Supplementary file 5 — Supplementary Table 1 [file 41416_2018_307_MOESM5_ESM.doc]

| **Table S1.** Patient's characteristics according to median CDA activity (cut-off 7.2 U/mg) | | | | | | | | | | | | |
| --- | --- | --- | --- | --- | --- | --- | --- | --- | --- | --- | --- | --- |
|  |  | CDA activity < 7.2 U/mg  N. (%) | | | |  | | CDA activity > 7.2 U/mg P (Fisher' Exact test)  N. (%) | | | |  |
|  |  |  | **61 pts** |  |  | | **60 pts** | |  |  |  | |
|  | Age, median yrs |  | 71 (49-81) |  |  | | 70 (45-87) | |  | 0.92 |  | |
|  | *Sex* |  |  |  |  | |  | |  |  |  | |
|  | Female |  | 13 (21.3) |  |  | | 14 (23.3) | |  | 0.82 |  | |
|  | Male |  | 48 (78.7) |  |  | | 46 (76.7) | |  |  |  | |
|  | ECOG PS |  |  |  |  | |  | |  |  |  | |
|  | 0 |  | 33 (54.1) |  |  | | 15 (25.0) | |  | 0.0027 |  | |
|  | 1 |  | 26 (42.6) |  |  | | 38 (63.4) | |  |  |  | |
|  | 2 |  | 2 (3.3) |  |  | | 7 (11.6) | |  |  |  | |
|  | Histology |  |  |  |  | |  | |  |  |  | |
|  | Adenocarcinoma |  | 16 (26.3) |  |  | | 12 (20.0) | |  | 0.70 |  | |
|  | Epidermoid |  | 37 (60.6) |  |  | | 38 (63.3) | |  |  |  | |
|  | Large cells |  | 8 (13.1) |  |  | | 10 (10.7 | |  |  |  | |
|  | Stage |  |  |  |  | |  | |  |  |  | |
|  | IIIB |  | 16 (26.2) |  |  | | 10 (16.7) | |  | 0.26 |  | |
|  | IV |  | 45 (73.8) |  |  | | 50 (83.3) | |  |  |  | |
|  | Therapy |  |  |  |  | |  | |  |  |  | |
|  | CDDP-Gem |  | 34 (55.7) |  |  | | 14 (23.3) | |  | 0.02 |  | |
|  | CBDCA-Gem |  | 27 (44.3) |  |  | | 46 (76.7) | |  |  |  | |
| CDDP: Cisplatin; CBDCA: carboplatin; GEM: gemcitabine; ECOG: Eastern Cooperative Oncology Group; PS: Performance Status | | | | | | | | | | | | |
