# Supplementary material for: Cytidine deaminase enzymatic activity is a prognostic biomarker in gemcitabine/platinum-treated advanced non-small-cell lung cancer: a prospective validation study
Source: Br J Cancer. 2018 Nov 8;119(11):1326–31. doi: 10.1038/s41416-018-0307-3 (PMC6265283; doi:10.1038/s41416-018-0307-3)
Supplement: Supplementary file 6 — Supplementary Table 2 [file 41416_2018_307_MOESM6_ESM.doc]

| **Table S2.** Patient's characteristics according to CDA activity (cut-off 8.35 U/mg) | | | | | | | | | |
| --- | --- | --- | --- | --- | --- | --- | --- | --- | --- |
|  |  | CDA activity < 8.35 U/mg | | |  | CDA activity > 8.35 U/mg P (Fisher' Exact test) | | |  |
|  |  |  | **74 pts** |  |  | **47 pts** |  |  |  |
|  | Age, median yrs |  | 70.5 (49-85) |  |  | 70 (45-87) |  | 0.86 |  |
|  | *Sex* |  |  |  |  |  |  |  |  |
|  | Female |  | 16 |  |  | 11 |  | 0.82 |  |
|  | Male |  | 58 |  |  | 36 |  |  |  |
|  | ECOG PS |  |  |  |  |  |  |  |  |
|  | 0 |  | 36 |  |  | 12 |  | 0.0054 |  |
|  | 1 |  | 36 |  |  | 28 |  |  |  |
|  | 2 |  | 2 |  |  | 7 |  |  |  |
|  | Histology |  |  |  |  |  |  |  |  |
|  | Adenocarcinoma |  | 19 |  |  | 9 |  | 0.55 |  |
|  | Epidermoid |  | 43 |  |  | 32 |  |  |  |
|  | Other histology |  | 12 |  |  | 6 |  |  |  |
|  | Stage |  |  |  |  |  |  |  |  |
|  | IIIB |  | 17 |  |  | 9 |  | 0.65 |  |
|  | IV |  | 57 |  |  | 38 |  |  |  |
|  | Therapy |  |  |  |  |  |  |  |  |
|  | CDDP-Gem |  | 36 |  |  | 12 |  | 0.013 |  |
|  | CBDCA-Gem |  | 38 |  |  | 35 |  |  |  |
| CDDP: Cisplatin; CBDCA: carboplatin; GEM: gemcitabine; ECOG: Eastern Coopererative Oncology Group; PS: Performance Status | | | | | | | | | |
