# Supplementary material for: Cytidine deaminase enzymatic activity is a prognostic biomarker in gemcitabine/platinum-treated advanced non-small-cell lung cancer: a prospective validation study
Source: Br J Cancer. 2018 Nov 8;119(11):1326–31. doi: 10.1038/s41416-018-0307-3 (PMC6265283; doi:10.1038/s41416-018-0307-3)
Supplement: Supplementary file 7 — Supplementary Table 3 [file 41416_2018_307_MOESM7_ESM.doc]

| **Table S3.** Multivariate analysis of CDA level on objective response (cut-off 7.2 U/mg) | | | | | | | | | |
| --- | --- | --- | --- | --- | --- | --- | --- | --- | --- |
|  | Effect |  | DF |  |  | Wald chi-square |  | Pr > Chi sqare |  |
|  | Age |  | 1 |  |  | 0.45 |  | 0.50 |  |
|  | Sex |  | 1 |  |  | 0.046 |  | 0.82 |  |
|  | ECOG PS |  | 2 |  |  | 2.38 |  | 0.30 |  |
|  | Histology |  | 2 |  |  | 7.56 |  | 0.022 |  |
|  | Stage |  | 1 |  |  | 2.0 |  | 0.15 |  |
|  | Type of platinum |  | 1 |  |  | 9.94 |  | 0.001 |  |
|  | CDA |  | 1 |  |  | 2.55 |  | 0.10 |  |
| ECOG: Eastern Coopererative Oncology Group; PS: Performance Status; CDA: Cytidine Deaminase | | | | | | | | | |
