# Supplementary material for: Cytidine deaminase enzymatic activity is a prognostic biomarker in gemcitabine/platinum-treated advanced non-small-cell lung cancer: a prospective validation study
Source: Br J Cancer. 2018 Nov 8;119(11):1326–31. doi: 10.1038/s41416-018-0307-3 (PMC6265283; doi:10.1038/s41416-018-0307-3)
Supplement: Supplementary file 8 — Supplementary Table 4 [file 41416_2018_307_MOESM8_ESM.doc]

| **Table S4.** Multivariate analysis of CDA level on objective response (cut-off 8.35 U/mg) | | | | | | | | | |
| --- | --- | --- | --- | --- | --- | --- | --- | --- | --- |
|  | Effect |  | DF |  |  | Wald chi-square |  | Pr>Chisquare |  |
|  | Age |  | 1 |  |  | 0.33 |  | 0.56 |  |
|  | Sex |  | 1 |  |  | 0.043 |  | 0.83 |  |
|  | ECOG PS |  | 2 |  |  | 2.29 |  | 0.31 |  |
|  | Histology |  | 2 |  |  | 8.23 |  | 0.016 |  |
|  | Stage |  | 1 |  |  | 2.43 |  | 0.11 |  |
|  | Type of platinum |  | 1 |  |  | 11.29 |  | 0.0008 |  |
|  | CDA |  | 1 |  |  | 4.12 |  | 0.04 |  |
| ECOG: Eastern Coopererative Oncology Group; PS: Performance Status; CDA: Cytidine Deaminase | | | | | | | | | |
