# Supplementary material for: Cytidine deaminase enzymatic activity is a prognostic biomarker in gemcitabine/platinum-treated advanced non-small-cell lung cancer: a prospective validation study
Source: Br J Cancer. 2018 Nov 8;119(11):1326–31. doi: 10.1038/s41416-018-0307-3 (PMC6265283; doi:10.1038/s41416-018-0307-3)
Supplement: Supplementary file 9 — Supplementary Table 5A-B [file 41416_2018_307_MOESM9_ESM.doc]

| **Table S5A.** Multivariate analysis of CDA level on Progression-free survival (cut-off 8.35 U/mg) | | | | | | | |
| --- | --- | --- | --- | --- | --- | --- | --- |
| Parameter DF Parameter Standard Chi-Square Pr > Chisq Hazard 95% Hazard Ratio Label  Estimate Error Ratio Confidence Limits | | | | | | | |
| Age | 1 | -0.01623 | 0.01491 | 1.1847 | 0.2764 | 0.984 | 0.956 1.013 |
| Sex Female | 1 | 0.55047 | 0.28231 | 3.8020 | 0.0512 | 1.734 | 0.997 3.016 sex Female |
| ECOG PS 1 | 1 | 0.50064 | 0.23169 | 4.6691 | 0.0307 | 1.650 | 1.048 2.598 PS 1 |
| ECOG PS 2 | 1 | 1.20025 | 0.46382 | 6.6964 | 0.0097 | 3.321 | 1.338 8.243 PS 2 |
| Stage IIIB | 1 | -0.49090 | 0.27448 | 3.1987 | 0.0737 | 0.612 | 0.357 1.048 Stage IIIB |
| CDA High > 8.35 | 1 | 0.47659 | 0.22985 | 4.2993 | 0.0381 | 1.611 | 1.026 2.527 CDA High > 8.35 |
| ECOG: Eastern Coopererative Oncology Group; PS: Performance Status; CDA: Cytidine Deaminase | | | | | | | |

| **Table S5B.** Multivariate analysis of CDA level on overall survival (cut-off 8.35 U/mg) | | | | | | | |
| --- | --- | --- | --- | --- | --- | --- | --- |
| Parameter DF Parameter Standard Chi-Square Pr > Chisq Hazard 95% Hazard Ratio Label  Estimate Error Ratio Confidence Limits | | | | | | | |
| Age | 1 | -0.00660 | 0.01480 | 0.1986 | 0.6559 | 0.993 | 0.965 1.023 |
| Sex Female | 1 | 0.19740 | 0.25126 | 0.6172 | 0.4321 | 1.218 | 0.744 1.993 sex Female |
| ECOG PS 1 | 1 | 0.68407 | 0.22429 | 9.3026 | 0.0023 | 1.982 | 1.277 3.076 PS 1 |
| ECOG PS 2 | 1 | 0.84754 | 0.42317 | 4.0114 | 0.0452 | 2.334 | 1.018 5.349 PS 2 |
| Stage IIIB | 1 | -0.93311 | 0.28568 | 10.6690 | 0.0011 | 0.393 | 0.225 0.689 Stage IIIB |
| CDA High > 8.35 | 1 | 0.82751 | 0.22529 | 13.4919 | 0.0002 | 2.288 | 1.471 3.558 CDA High > 8.35 |
| ECOG: Eastern Coopererative Oncology Group; PS: Performance Status; CDA: Cytidine Deaminase | | | | | | | |
